# Evaluating the Quantitative Accuracy and Application of DNA Metabarcoding for Dietary Reconstruction in Ruminants

**DOI:** 10.1002/ece3.72878

**Published:** 2026-01-14

**Authors:** Hannah Vallin, Mariecia Fraser, Robin J. Pakeman, Helen Hipperson

**Affiliations:** ^1^ Institute of Biological, Environmental and Rural Sciences (IBERS) Aberystwyth University Aberystwyth UK; ^2^ Pwllpeiran Upland Research Centre, IBERS Aberystwyth University Aberystwyth UK; ^3^ Ecological Sciences, the James Hutton Institute Aberdeen UK; ^4^ School of Biosciences University of Sheffield Sheffield UK

**Keywords:** diet composition, DNA metabarcoding, grazing management, herbivore

## Abstract

DNA metabarcoding offers a powerful, non‐invasive tool to identify dietary composition with high taxonomic resolution, yet its quantitative accuracy and bias remain a well‐recognised limitation across taxa and sample types. This universal challenge is particularly evident in herbivores, where plant material introduces additional amplification constraints. This study evaluates the accuracy of DNA metabarcoding in reconstructing the diets of sheep under controlled feeding trials involving high and low digestibility forage, using two widely used plant DNA barcodes (ITS2 and *trn*L). A secondary trial tested the detectability and proportional representation of a target species, *Medicago sativa* , when added to the diet in varying amounts (1%, 5%, 10%). ITS2 provided greater species‐level resolution, while trnL showed broader taxonomic coverage but reduced precision. Both markers distinguished diet treatments effectively; however, faecal DNA showed proportional discrepancies from vegetation input, particularly under low‐digestibility conditions. *M. sativa* was reliably detected even at 1% inclusion but was consistently overrepresented in sequence reads. Our findings highlight the strengths and limitations of DNA metabarcoding for herbivore diet studies and underscore the importance of marker choice and the effects of differential digestion biases. These findings demonstrate the need for multi‐marker approaches and calibration controls in dietary studies, especially when quantitative interpretation is required. Despite limitations in quantitative accuracy, faecal DNA metabarcoding provides valuable insights into herbivore diet composition and preferences, with future refinements expected to improve its resolution and reliability for ecological monitoring and grazing management.

## Graphical abstract

This study assessed the accuracy of DNA metabarcoding using ITS2 and trnL markers to reconstruct sheep diets under controlled feeding trials with high‐ and low‐digestibility forage, and in a secondary trial with low‐level inclusions of *Medicago sativa* . ITS2 offered higher species‐level resolution, while trnL provided broader taxonomic coverage but less precision when distinguishing diet treatments; moreover, faecal DNA composition diverged from vegetation inputs under low‐digestibility conditions. *M. sativa* was detected at 1% inclusion but overrepresented in sequence reads, highlighting the need for multi‐marker approaches, curated reference libraries, and calibration controls when interpreting herbivore dietary data quantitatively.

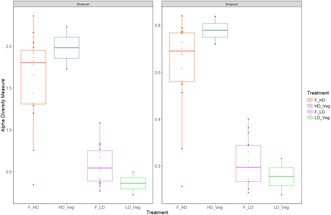

## 1 Introduction

Increasing pressures from climate change, habitat loss, and shifts in resource quality and availability are intensifying challenges faced by grazing herbivores (Pansu et al. 2022; Serrouya et al. 2021; Wang et al. 2025). These pressures can lead to fragmented habitats and limited resource access, ultimately affecting individual health and welfare, further influencing survival, reproduction, and population dynamics (Kowalczyk et al. 2011). Moreover, herbivores grazing in inappropriate habitats or at unsustainable densities can negatively impact habitats and other species, underlining the importance of aligning herbivore management with broader ecosystem conservation goals (Fraser et al. 2022). Understanding dietary composition provides valuable insights into herbivores' foraging ecology, plant‐herbivore interactions, and how the use of available resources shapes feeding behaviour and habitat selection.

Historically, a variety of methods have been employed to determine herbivore diets, such as rumen sampling, n‐alkane markers, stable isotope analysis, visual grazing observations, and microhistology (Grant et al. 1985; Dove and Mayes 1991; Garnick et al. 2018). While stable isotope and n‐alkane analyses are well established, cost‐effective, and scalable, they offer limited taxonomic resolution and cannot easily distinguish among the multiple plant species typically consumed by herbivores. In contrast, morphology‐based approaches such as microhistology and rumen content analysis are time‐consuming and require expert plant identification, constraining both taxonomic precision and throughput (Holechek et al. 1982; Garnick et al. 2018; Mayes and Dove 2000; Scasta et al. 2019). Over the last decade, advances in DNA metabarcoding and high‐throughput next‐generation sequencing platforms have revolutionised dietary studies by improving the speed, accuracy, and taxonomic resolution of DNA‐based dietary analyses (Alberdi et al. 2019; Lamb et al. 2019; Sousa et al. 2019). This approach allows for the simultaneous identification of multiple species within multiple complex biological samples, such as faeces or stomach contents for dietary studies, and can effectively address many of the limitations inherent in earlier methods (Deagle et al. 2010; Pompanon et al. 2012). DNA metabarcoding can provide more detailed species composition of diets compared to alternative methods, and with relatively low costs and rapid sample turnaround (Alberdi et al. 2019; Lamb et al. 2019). Furthermore, DNA metabarcoding enables targeted sequencing of specific DNA, such as plant material, using taxon‐specific markers (Johnson et al. 2023). Faecal DNA (fDNA) metabarcoding has emerged as a non‐invasive, rapid molecular technique and has proven highly advantageous for large‐scale diet investigations across a wide range of taxa, including insects, amphibians, reptiles, fish, mammals, and birds (e.g., Deagle et al. 2010; Kowalczyk et al. 2011; Murano et al. 2023; Zinger et al. 2019). Herbivore‐focused research has more than doubled since the early 2000s, with fDNA determining botanical composition of diets in both wild and domesticated free‐ranging herbivores (Garnick et al. 2018; Kamenova, Meyer and Brysting, et al. 2024; Scasta et al. 2019).

Despite the advantages of DNA metabarcoding, significant challenges remain in accurately quantifying dietary components, particularly in herbivores, where mixed‐plant diets and variable chloroplast DNA content introduce additional bias (Iwanowicz et al. 2016; Lamb et al. 2019; Sousa et al. 2019). While quantitative inaccuracy is a broader issue across dietary and environmental metabarcoding studies (e.g., Sato 2024), these challenges are especially pronounced in herbivore systems due to the complex and heterogeneous nature of vegetation consumed. One potential complication is the variability of plastid numbers and genome size between plant species, which can cause over‐ and under‐representation in sequence counts, leading to discrepancies between DNA reads and the actual biomass consumed (Deagle and Tollit 2007; Taberlet et al. 2018; Piñol et al. 2018). However, while such copy‐number variation is frequently highlighted as a possible source of bias, its influence on diet metabarcoding remains largely untested. Recent work (Moinard et al. 2023) has proposed methods to correct for these biases by combining “standard quantitative PCR techniques (qPCR and digital droplet PCR) with a realistic stochastic model of PCR dynamics that accounts for PCR saturation”, though further validation is still required. Plant DNA metabarcoding also presents specific challenges related to marker selection, as no single barcode region achieves both universal amplification and high taxonomic resolution across all plant groups (Coissac et al. 2012; Ficetola et al. 2010; Taberlet et al. 2018). Furthermore, factors such as differential digestion rates of plant tissues and variation in plastid content among species can influence DNA recovery from faecal samples, adding complexity to dietary analyses (Nakahara et al. 2016; Willerslev et al. 2014; Lamb et al. 2019). More digestible and chlorophyll‐rich material can be underrepresented in faecal DNA due to extensive degradation during digestion, especially when targeting longer DNA fragments (Kamenova et al. 2018; Stapleton et al. 2022). Conversely, fibrous or lignified tissues may be relatively overrepresented, as they yield more persistent DNA fragments. Additionally, variation in bioinformatic filtering thresholds (e.g., minimum read counts or clustering criteria) and incomplete global reference sequence libraries can influence which taxa are retained or discarded during data processing, thereby shaping estimates of species richness and composition (Dunn et al. 2018; Iwanowicz et al. 2016; Mallott et al. 2018; Vallin et al. 2025). In this study, we developed a curated reference library with the aim to improve the accuracy of taxonomic assignment for the sown plant species and to reduce the number of unclassified reads. This highlights the importance of using standardised yet flexible bioinformatic pipelines and comprehensive local reference databases to ensure robust interpretation of dietary metabarcoding data.

The selection of genetic markers used in metabarcoding also plays a crucial role in determining the accuracy and taxonomic resolution of species identification. Commonly used plant markers, such as *trnL*, *rbcL*, *matK*, and ITS regions, each have strengths and limitations. The ITS2 region offers high taxonomic resolution; however, its applicability can be limited across certain plant taxa due to amplification biases, intraspecific variation, and incomplete reference databases (Yao et al. 2010). The trnL intron, which includes both conserved and variable regions, is well suited for analysing degraded DNA but generally provides lower species‐level resolution due to limited sequence variability (Sato 2024; Taberlet et al. 2007). In contrast, *rbcL* is easily amplified and effective for higher‐level taxonomic classification, while *matK* offers higher variability but produces longer amplicons that are difficult to recover from degraded faecal DNA and challenging to sequence on short‐read platforms such as Illumina (Espinosa Prieto et al. 2024; Moorhouse‐Gann et al. 2018). The choice of marker therefore represents a trade‐off between fragment length, taxonomic discrimination ability, and amplification success. In the context of metabarcoding, combining markers can expand taxonomic coverage and improve quantitative performance, but also introduces comparability issues between datasets generated with different primer sets (Chen et al. 2009; Sousa et al. 2019; Tercel et al. 2021; Valentini, Miquel, et al. 2009; Valentini, Pompanon, and Taberlet 2009).

Despite its challenges, DNA metabarcoding has great potential for analysing herbivore diets, particularly when validated through controlled feeding trials that compare known diets with sequencing data (Deagle et al. 2010; Scasta et al. 2019). These trials are essential for addressing challenges unique to herbivores, such as differential digestion of plant material (Willerslev et al. 2014; Nakahara et al. 2016). Improving experimental design remains essential to reduce biases across the workflow and improve quantitative interpretation of sequence data. Quantitative bias in DNA metabarcoding remains a major constraint across taxa (Sato 2024; Stapleton et al. 2022), driven by factors such as primer bias, sequence copy number variation, and differential amplification efficiency. In herbivores, these issues are often compounded by the complex and heterogeneous nature of plant material consumed (Guo et al. 2018). Previous studies advocate the need to incorporate prior taxonomic knowledge to improve primer choice and taxonomic assignment (Sousa et al. 2019), as well as developing accurate reference barcode libraries and using mock community samples to support more quantitative interpretations (Thomas et al. 2014). While DNA metabarcoding has been widely applied to diet analyses in wild herbivores, relatively few studies have experimentally tested its accuracy under controlled feeding conditions, particularly in species with diverse, mixed diets (Thomas et al. 2014; Willerslev et al. 2014; Nakahara et al. 2016; Scasta et al. 2019).

The primary objective of this study was to evaluate the accuracy of DNA metabarcoding in estimating the proportional plant species composition in herbivore diets by testing two contrasting sheep diets, high and low digestibility, to evaluate how species‐specific biases may affect sequence results for both major and minor dietary components under controlled conditions. Additionally, we investigated the method's ability to detect and quantify small, known proportions (1%, 5%, and 10%) of a target species, *Medicago sativa* , passing through the digestive tract in the faeces. Using faecal DNA (fDNA) metabarcoding in combination with a curated, study‐specific reference library, we applied two genetic markers (trnL and ITS2) to enhance species‐level resolution and evaluate how well metabarcoding data reflect known plant composition derived from traditional botanical sorting (manual separation and morphological identification of plant species within vegetation samples). Our study focuses on assessing the quantitative reliability of fDNA metabarcoding for reconstructing herbivore diets across different forages and digestibility levels, acknowledging that both molecular and morphological approaches have intrinsic biases affecting proportional representation.

## 2 Methods

All feeding trials were conducted in accordance with the UK Animals (Scientific Procedures) Act 1986, with approval from the Aberystwyth University Animal Welfare and Ethical Review Board. Feeding trials took place at Pwllpeiran Research Centre, Aberystwyth University, using Soay sheep (wethers) to evaluate the accuracy of DNA metabarcoding in reconstructing herbivore diets.

### 2.1 Experimental Design

#### 2.1.1 Controlled Feeding Trials

##### 2.1.1.1 Trial 1—Digestibility

This study consisted of two experimental runs conducted between July and September 2019. Sixteen Soay wethers were fed two contrasting diets: (1) a high‐digestibility (HD) grass/legume mixture and (2) a low‐digestibility (LD) *Molinia caerulea* ‐dominated pasture. The goal was to test the ability of DNA metabarcoding to reconstruct diet composition across these digestibility contrasts.

#### 2.1.2 Diet Preparation and Feeding

High‐digestibility forage was harvested from a grass/legume mixed sward, while low‐digestibility forage was sourced from semi‐natural *Molinia*‐dominated grassland. All sheep underwent an 8‐day adaptation period followed by 5 days of feeding the respective diets. Forage was weighed according to maintenance requirements (AFRC 1993), and faecal samples were collected during the last two days of the trial (further details in Data S1: Section 1).

##### 2.1.2.1 Trial 2—Known Diet Proportions

Conducted in October 2019 at the Pwllpeiran Research Centre, this trial involved 12 adult Soay wethers (> 2 years old) housed under the same controlled indoor conditions described for Trial 1. Animals were randomly assigned to three diet groups (*n* = 4 per group), each receiving 1%, 5%, or 10% *Medicago sativa* (lucerne) on a dry‐matter (DM) basis within a basal diet of *Beta vulgaris* pellets and mixed chopped spring oat ( *Avena sativa* ) and barley straw ( *Hordeum vulgare* ). The aim was to quantify the ability of DNA metabarcoding to detect low‐level inclusion of a target species within a controlled diet. Diet Preparation and Feeding: Fresh *M. sativa* was harvested from experimental plots, DM content determined, and incorporated into the basal diet according to individual maintenance requirements (AFRC 1993). All components were weighed separately on a DM basis for each animal and fed in two equal meals daily (08:00 and 16:00). Diets were not fed ad libitum to prevent selective feeding; refusals were collected and weighed daily. Sheep underwent a 14‐day adaptation period, during which they were gradually transitioned from pasture to the basal diet and acclimatised to individual pen housing, ensuring rumen microbial adjustment to the experimental diet. The experimental diets were then fed for 5 days, with faecal samples collected during the final two days (further details in Data S1: Section 2).

### 2.2 Sample Collection

#### 2.2.1 Forage Sampling

For both trials, forage dry matter (DM) content was measured at harvest by oven drying sub‐samples (100 g) for 24 h at 60°C and re‐weighing. Any refusals were collected daily, weighed, and recorded to keep records of total dietary intake and ensure all individuals were consuming their allocated diet. During the experimental period, sub‐samples (100 g) of feed as offered were collected for each dietary component separately and stored at −80°C for subsequent DNA extraction and metabarcoding.

#### 2.2.2 Faecal Sampling

Faecal samples were collected from individually penned sheep, ensuring that each sample could be assigned to a specific animal. Pens were checked twice daily during the final two days of each trial, and only freshly deposited droppings were collected. Fresh pellets were identified by their warmth, moisture, and appearance, and previously collected material was removed immediately after sampling to avoid repeat collection or cross‐contamination. All samples were placed into individually labelled plastic bags, crudely homogenised, weighed, rolled to exclude air, and stored at −80°C until DNA extraction and metabarcoding analysis.

### 2.3 DNA Extraction

#### 2.3.1 Faecal DNA Extraction

For both trials, faecal DNA was extracted using the QIAamp PowerFaecal Pro DNA Kit (Qiagen) following the manufacturer's protocol. To account for the large mass of sheep faeces, multiple faecal pellets from each individual were homogenised to ensure a representative and uniform sample. Approximately 250 mg of homogenised material was used per extraction, with two independent extractions performed for each sample and subsequently pooled in the final elution buffer to maximise DNA yield and minimise potential bias from heterogeneous material. Homogenised faecal samples were disrupted using a TissueLyser II (Qiagen) at 25 Hz for 5 min, re‐oriented at 180 degrees, and shaken again for 5 min at speed 25 Hz. All DNA extracts were subsequently quantified on a spectrometer (Epoch Microplate Spectrophotometer, BioTech). All samples were stored at −20°C until polymerase chain reaction (PCR) was performed.

#### 2.3.2 Vegetation DNA Extraction

Vegetation samples were processed similarly, with frozen samples homogenised in liquid nitrogen before extraction using the QIAamp PowerFaecal Pro DNA Kit. DNA quantity was assessed as described above before further analysis.

### 2.4 PCR Amplification and Sequencing

#### 2.4.1 Trial 1—Digestibility

The complete second internal transcribed spacer of nuclear ribosomal DNA (ITS2) (fragment size ~320 bp) (Ranieri et al. 2025) was amplified using the primer pair ITS2F (ATGCGATACTTGGTGTGAAT) (Chen et al. 2010), and UniPlantR (CCCGHYTGAYYTGRGGTCDC) (Moorhouse‐Gann et al. 2018), along with the P6 loop of the plastid trnL (UAA) region (~147 bp) (Ranieri et al. 2025), using the trnL‐c (CGAAATCGGTAGACGCTACG) and trnL‐h (CCATTGAGTCTCTGCACCTATC) primers (Taberlet et al. 2007). Both markers were amplified across all samples following a two‐step PCR protocol (Data S1: Section 3). PCR products were purified using ProNex beads (Promega, Madison, WI, USA) and quantified using 2 μL of PCR product on a fluorimeter to check the DNA concentration before pooling for Illumina MiSeq sequencing, carried out at the Genomics & Bioinformatics Core Facility at the University of Edinburgh, using an Illumina MiSeq V2 500 cycle sequencing kit and generating 2 × 210 bp paired reads as appropriate for the amplicon lengths.

#### 2.4.2 Trial 2—Known Diet Proportions

The chloroplast trnL intron was amplified using the primer pair outlined above but with a slightly different library preparation protocol. Custom dual indices (short barcodes 8 base pairs long) were added to both the forward and reverse primers to serve as molecular tags to be able to identify each sample from the pooled sequencing run. Using a combination of 12 forward and 8 reverse barcodes allowed for different combinations within a 96‐well plate, creating a unique combination for each sample (barcode and adaptor sequences provided in Data S1: Section 4). PCR products were cleaned, quantified, and sequenced via Illumina MiSeq at the Genomics & Bioinformatics Core Facility at the University of Edinburgh, using an Illumina MiSeq V2 500 cycle sequencing kit and generating 2 × 210 bp paired reads, as appropriate for the amplicon lengths.

Both projects incorporated mock community samples to assess sequencing accuracy. These comprised equal proportions of DNA from five known tropical plant species sourced from pre‐existing extracts supplied by the National Botanic Garden of Wales (see Data S1: Section 5). Tropical species were selected to ensure that none overlapped with taxa present in the feeding trials, thereby avoiding cross‐sample contamination or misassignment. The mock communities showed a strong match between expected and observed taxa, with all species detected and no non‐target sequences recovered.

### 2.5 Bioinformatics and Sequence Processing

All sequence data were processed using the DADA2 package (Callahan et al. 2016) (v1.28.0) in R (v4.3.1), run on the University of Sheffield's High Performance Computing cluster ‘Bessemer’. Primary quality control and filtering were performed using the DADA2 pipeline following established best‐practice guidelines. Raw sequence reads were initially assessed using per‐base quality score plots to evaluate read quality profiles and identify low‐quality regions. Reads containing ambiguous or unknown bases (Ns) were removed. Primer sequences were then identified and trimmed using cutadapt (Martin 2011) prior to further processing. Based on inspection of the sequence quality profiles, read truncation lengths of 150 bp for trnL and 220 bp for ITS2 were applied. Sequence reads were then dereplicated to collapse identical reads into unique sequences, after which paired‐end reads were merged. Chimeric sequences were identified and removed using the DADA2 consensus method. Amplicon sequence variants (ASVs) were inferred using the error model learned from the data.

A study‐specific, regionally restricted reference database was constructed for trnL and ITS2 by extracting plant sequences from the NCBI nucleotide database using updated search terms following Richardson et al. (2020). Accession numbers were processed using the R package Taxonomizr (Sherrill‐Mix 2021) to assign complete taxonomic lineages. Sequences were then filtered based on a compiled list of 203 dietary plant species derived from vegetation surveys of the study sites and plant components used in the feeding trials. Target barcode regions were extracted using MetaCurator (Richardson et al. 2020), guided by representative amplicon sequences aligned in Geneious Prime version 2019.0.4 (Biomatters Ltd.). This filtering approach restricted taxonomic assignment to taxa known to occur within the study system, reducing spurious assignments while recognising that the reference database relies on publicly available sequences and does not represent a voucher‐curated library. Sequences corresponding to the five exotic plant species included in the mock community were incorporated into the reference database from validated NCBI accessions and treated separately from the regional species list for validation purposes only.

Taxonomic assignments for each ASV were performed in DADA2 using the assignTaxonomy function, which applies a naïve Bayesian classifier to compare ASV sequences against the study‐specific, regionally restricted reference database for each marker. Taxonomic assignments were made hierarchically from kingdom to species level, with confidence determined by bootstrap resampling of Kmers within the classifier. Only taxonomic assignments exceeding the minimum bootstrap confidence threshold recommended in DADA2 (default ≥ 50%) were retained, and ASVs failing to meet this threshold were assigned to the highest supported taxonomic rank. Species‐level assignments were accepted only where sequences matched reference entries unambiguously and above the confidence threshold; otherwise, assignments were restricted to genus or family level as appropriate. A matrix of ASV sequence counts per sample was then exported from DADA2, and ASV counts were transformed to relative abundance for downstream analyses.

### 2.6 Statistical Analysis

Following taxonomic assignment, data processing was conducted using the *phyloseq* package (McMurdie and Holmes 2013) in R version 4.3.1 (R Core Team 2023). All blank negative controls containing no sequences were removed from further analysis. Rarefaction curves were generated to assess coverage quality for each marker, with samples containing fewer than 5000 reads subsequently excluded. Alpha diversity metrics (Shannon and Simpson indices) were calculated and compared between groups using the Wilcoxon rank sum test, with visualisations created using ggplot2 (Wickham 2014). Using functions from the *vegan* package in R (Oksanen et al. 2013), non‐metric multidimensional scaling (nMDS) with Bray–Curtis dissimilarity was then used to visualise taxonomic composition differences between diets, and a principal coordinate analysis (PCoA) further explored patterns within the data.

To test for significant differences between diets, a permutational multivariate analysis of variance (PERMANOVA) was conducted using the *adonis2* function in the *vegan* package, followed by a pairwise PERMANOVA to evaluate treatment, sampling date, and individual sheep as predictors of taxonomic composition. Additionally, a similarity percentage (SIMPER) analysis in PAST 4 (Hammer et al. 2001) identified taxa contributing most to observed dissimilarities between diets, particularly highlighting major component species (Clarke 1993). To confirm these differences, a one‐way ANOVA on individual species was also performed.

Taxa bar plots at family, genus, and species levels were generated using relative abundance data from each gene, with minor taxa aggregated for clarity. Differential abundance analysis was conducted using the DESeq2 package (Love et al. 2014) and the *exacttest* function from EdgeR to identify significantly differentially abundant taxa between diets.

## 3 Results

### 3.1 Trial 1 – Digestibility

#### 3.1.1 Sequencing Data

Sequencing generated 1,809,227 raw reads for ITS2 and 979,560 for trnL. After filtering, a total of 887,146 reads for ITS2 and 697,859 for trnL were retained across 54 samples. The ITS2 marker generated 2997 amplicon sequence variants (ASVs), with taxonomic assignments at the family (64%), genus (63%), and species (60%) levels. Meanwhile, trnL produced 745 ASVs, with assignments at the family (61%), genus (52%), and species (44%) levels.

All negative control samples contained no sequence reads after quality filtering and were removed from further analysis. Mock community samples showed high concordance with the expected composition, with all target taxa recovered and no unexpected taxa detected, and were therefore excluded from downstream analyses.

Sequencing analysis of both ITS2 and trnL markers yielded distinct yet complementary perspectives on dietary composition, as each marker recovered partially overlapping but non‐identical sets of taxa. ITS2 detected a greater number of taxa overall (69 taxa across 16 families), providing higher species‐level resolution, while trnL captured a slightly broader range of families (45 taxa across 18 families), including taxa not detected by ITS2.

#### 3.1.2 Alpha Diversity and Sample Clustering

**FIGURE 1:**
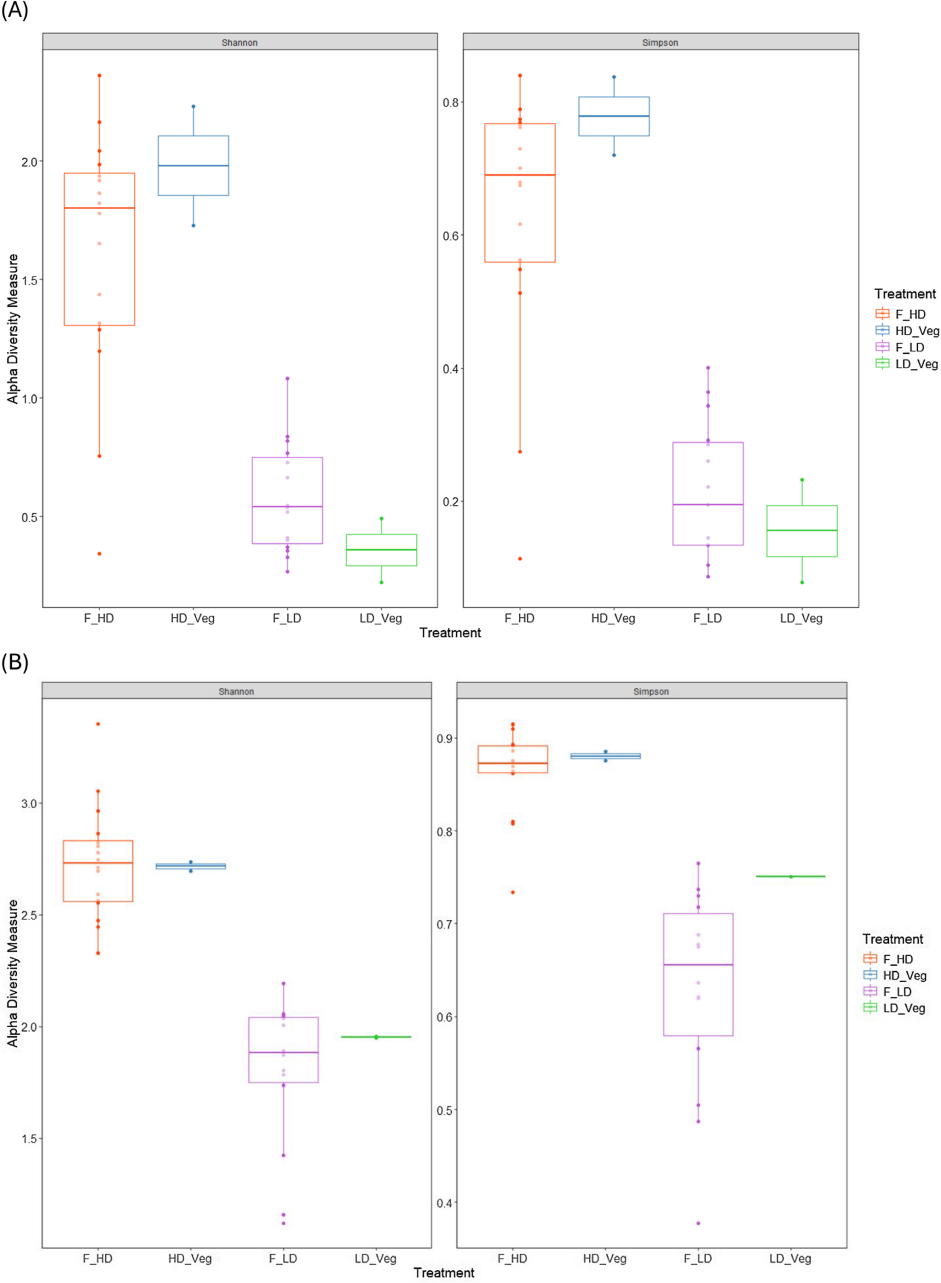
Alpha diversity box plot for the Shannon and Simpson diversity metrics from the ITS2 dataset (A) and trnL dataset (B). Treatments as follows: F_HD, high digestibility diet faecal samples; F_LD, Low digestibility diet faecal samples; HD Veg, high digestibility diet corresponding veg samples; LD Veg, low digestibility diet corresponding veg samples.

**FIGURE 2:**
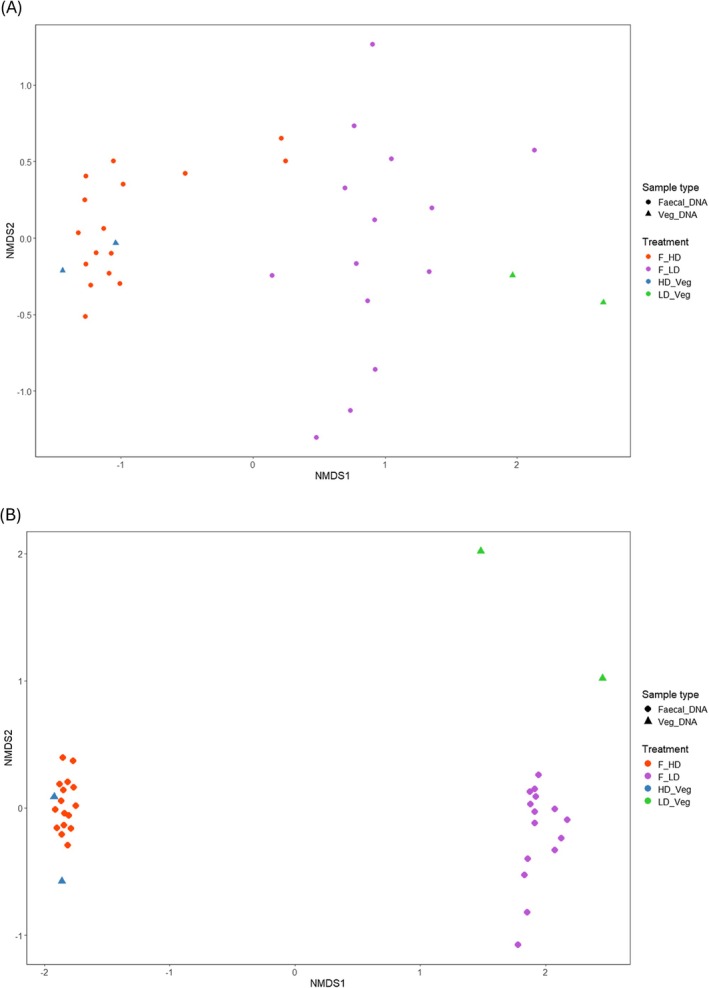
Non‐metric multidimensional scaling (nMDS) ordination on Bray‐Curtis index of amplicon sequence variants (ASV) from ITS2 dataset (A) and the trnL dataset (B).

Alpha diversity metrics (Shannon and Simpson indices) were calculated for both markers, revealing a higher diversity within the high‐digestibility (HD) diet samples compared to the low‐digestibility (LD) samples for both faecal and vegetation DNA. Although faecal samples from the LD treatment (F_LD) showed marginally higher diversity than corresponding vegetation samples (LD_Veg), this likely reflects the cumulative nature of faecal DNA, which integrates multiple feeding events and can detect trace or incidentally ingested taxa not observed in the botanical separations (Figure 1). Non‐metric multidimensional scaling (nMDS) revealed consistent separation between HD and LD samples for both ITS2 and trnL markers, with clear clustering of HD faecal and HD vegetation samples, and distinct patterns also observed for LD samples (Figure 2). These differences were statistically supported by PERMANOVA, with significant effects of treatment for both ITS2 (*p* = 0.001) and trnL (*p* = 0.001). Pairwise comparisons revealed a significant divergence between LD faecal and LD vegetation samples (*p*.adj < 0.015) for both markers, indicating that the composition of the low‐digestibility experimental diet, as inferred from sequenced vegetation subsamples and botanical separations, was not fully reflected in the faecal DNA under low‐digestibility conditions.

#### 3.1.3 Taxa Detection and Resolution

**FIGURE 3:**
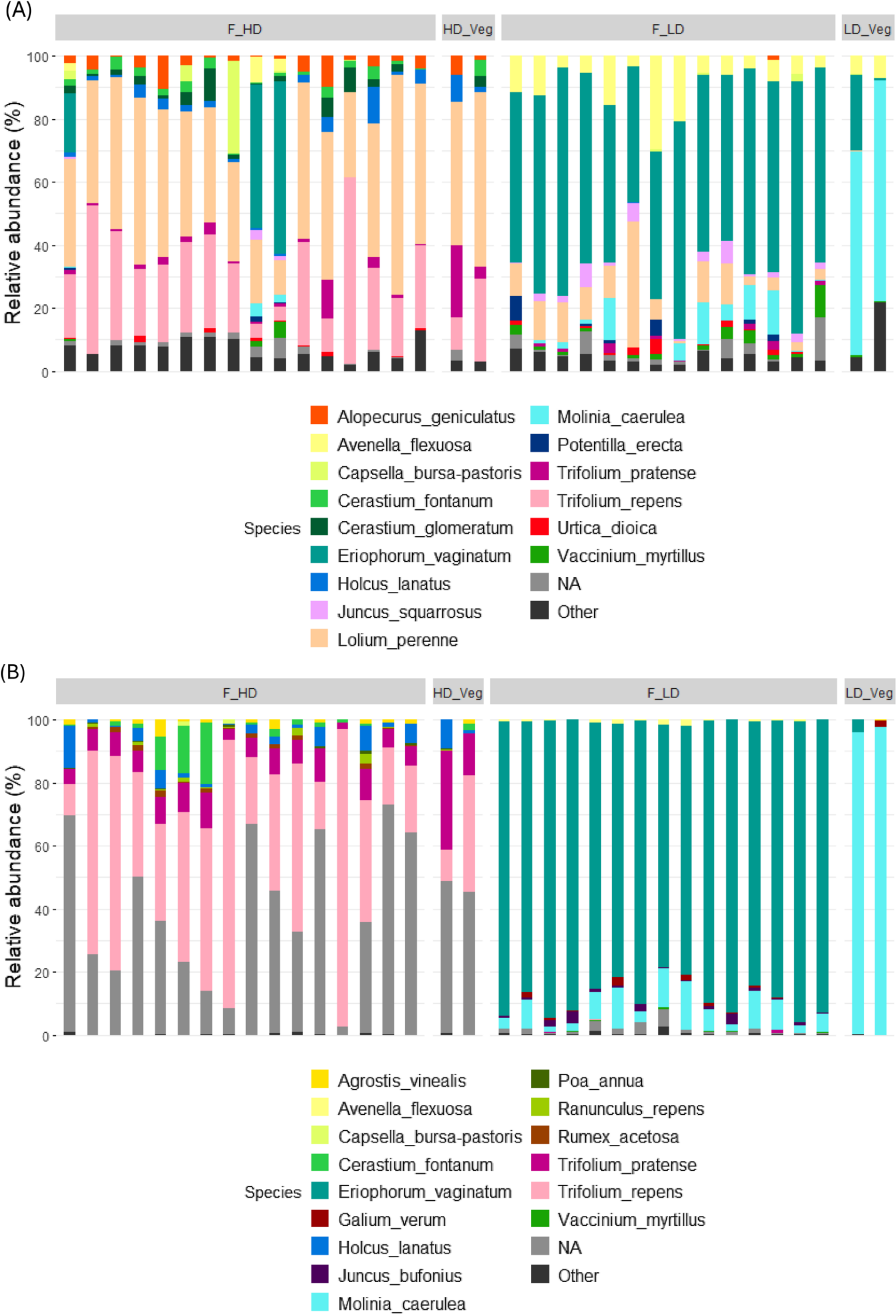
Taxa bar plot to show relative abundance (%) of the most abundant species (top 15) within each treatment. F_HD, high digestibility diet faecal samples; F_LD, low digestibility diet faecal samples; HD Veg, high digestibility diet corresponding veg samples; LD Veg, low digestibility diet corresponding veg samples from ITS2 dataset (A) and the trnL dataset (B).

The ITS2 marker distinguished clearly between the high‐digestibility (HD) and low‐digestibility (LD) experimental diets. In the HD diet, taxa including *Lolium perenne* , *Trifolium* spp., *Alopecurus geniculatus* , *Holcus lanatus* , and *Cerastium* spp. dominated the metabarcoding profiles (Figure 3A). Additional taxa such as *Eriophorum vaginatum* , *Molinia caerulea* , and *Juncus squarrosus* were also detected in a subset of HD faecal samples.

Botanical separation based on hand‐sorted vegetation samples (Table 1) showed broadly similar species presence to the ITS2 metabarcoding results for the HD diet. However, this comparison was descriptive, as formal statistical testing of diet composition or diversity between botanical and faecal samples was not undertaken due to the limited number of sequenced replicates per experimental diet. Consequently, while metabarcoding recovered many of the dominant dietary taxa, the results should be interpreted as qualitative agreement rather than a quantitative assessment of dietary accuracy.

**TABLE 1:** List of component species identified from botanical separations of experimental diet 1, high digestibility grass forage and legume mixture.

Species	Functional group	DM%	DM yield	SD
*Trifolium repens*	Legume	15.657	12.740	3.861
*Lolium* spp.	Graminoid	17.057	11.000	1.428
*Holcus lanatus*	Graminoid	21.089	3.505	7.128
*Trifolium pratense*	Legume	16.404	1.061	2.270
*Cerastium fontanum*	Forb	22.384	0.963	1.881
*Rumex*	Forb	17.683	0.458	0.057
*Ranunculus repens*	Forb	13.380	0.432	1.209
*Taraxacum* spp.	Forb	18.902	0.078	0.071
*Poa* spp.	Graminoid	21.765	0.019	0.092
*Urtica* spp.	Forb	50.000	0.003	0.007
Unidentified leaves	Unidentified leaves	21.563	0.035	0.226

*Note:* Average weights provided from two experimental sample dates. DM (Dry matter (g)).

In contrast, the LD diet (Table 2) was less diverse, with faecal samples dominated by *Eriophorum vaginatum, M* *. caerulea* , *L* *. perenne* , *J. squarrosus*, and *Avenella flexuosa* (Figure 3B). The LD vegetation samples were primarily composed of *M. caerulea* due to the *Molinia*‐dominated grassland source. However, *E. vaginatum* was more dominant in the faecal samples, leading to notable differences between LD faecal and vegetation samples.

**TABLE 2:** List of component species identified from botanical separations of experimental diet 2, low‐digestibility hill grass Molina mixture.

Species	Functional group	DM%	DM yield	SD
*Molinia caerulea*	Graminoid	53.796	51.020	13.944
*Festuca Ovina*	Graminoid	65.950	7.980	6.039
*Eriophorum vaginatum*	Graminoid	77.358	0.205	0.332
*Sphagnum* spp.	Bryophyte	64.646	0.320	0.021
*Galium saxatile*	Forb	12.500	0.005	0.057

*Note:* Average weights provided from two experimental sample dates.

**FIGURE 4:**
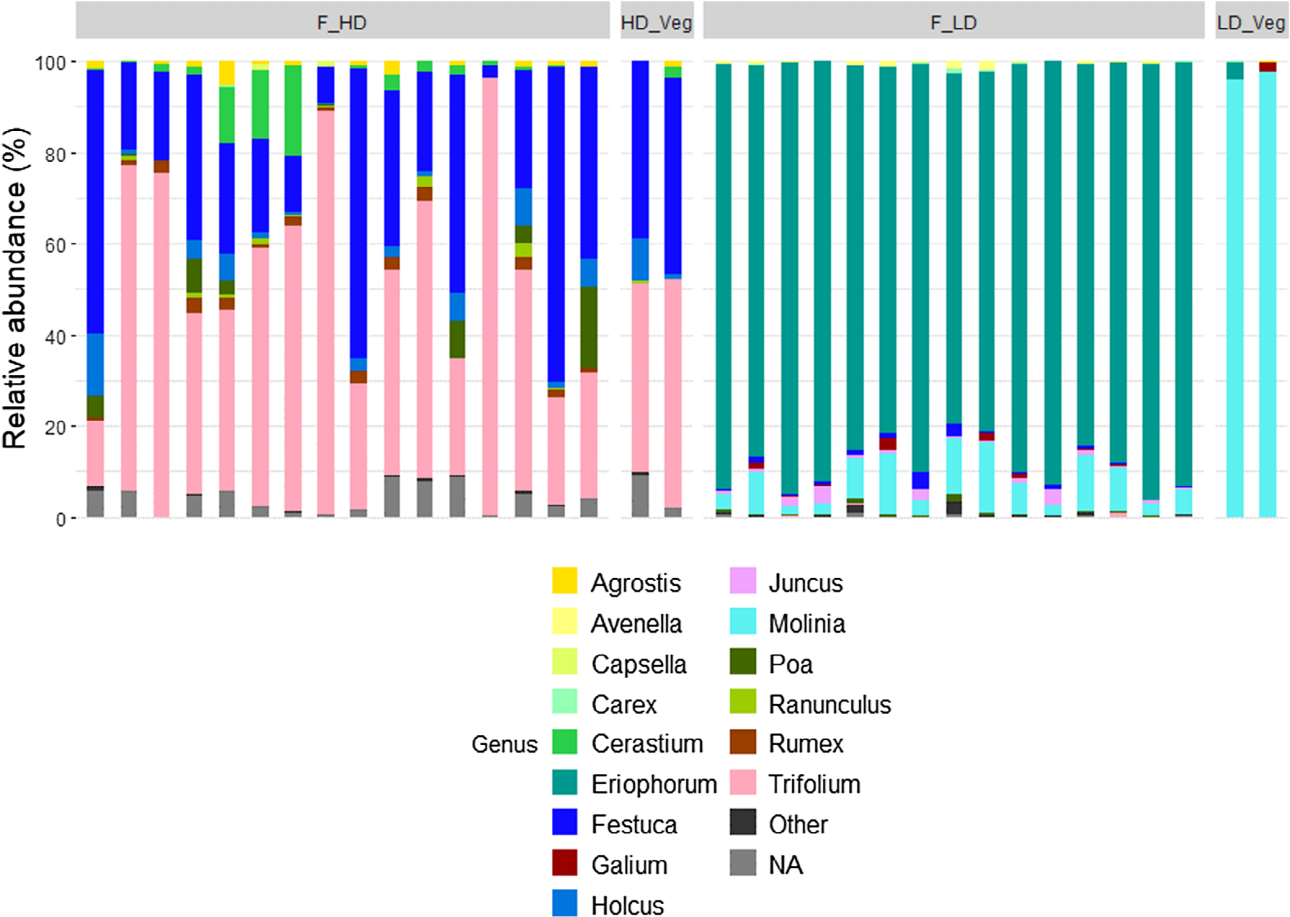
Taxa bar plot to show relative abundance (%) of the most abundant Genera within each treatment for the trnL dataset. HD, high digestibility diet faecal samples; HD Veg, high digestibility diet corresponding veg samples; LD, low digestibility diet faecal samples; LD Veg, low digestibility diet corresponding veg samples.

Similarly, the trnL marker distinguished between HD and LD diets, though with limitations in taxonomic resolution. A substantial proportion of ASVs from HD samples could not be resolved to species level and were therefore classified as “NA” at that rank, but were successfully assigned at higher taxonomic levels, primarily genus or family. Notably, trnL did not resolve *L. perenne* , a major component of the HD diet based on botanical records, with Poaceae taxa instead commonly grouped at the genus level (e.g., *Festuca* spp.), highlighting limited species‐level discrimination within this family (Figure 4).

In both the HD and LD diets, trnL results paralleled the ITS2 dataset in that HD faecal samples mirrored the HD vegetation composition well. However, as with ITS2, there was a discrepancy between LD faecal and vegetation samples, with *E. vaginatum* unexpectedly dominating LD faecal samples and an underrepresentation of *M. caerulea* in the faecal DNA samples compared to the veg DNA (Figure 3B). This finding aligns with ITS2 observations, suggesting that both markers reveal similar patterns of diet composition, though ITS2 consistently provided higher taxonomic resolution.

#### 3.1.4 Dissimilarity and Major Taxa Contributors

The SIMPER analysis for both ITS2 and trnL markers identified key taxa that contributed most to the dissimilarity between HD and LD diets (Tables 3, 4, 5), with notable consistency between markers. However, the more biologically relevant comparison lies in the differences between faecal and vegetation samples particularly for the LD diet (Table 4). For both markers, *E. vaginatum* (a dominant species in the LD diet) significantly contributed to the observed dissimilarities, along with *T. repens* and *L. perenne* in the HD diet (Table 3). The ANOVA confirmed significant differences (*p* < 0.05) for the primary contributing species in both datasets, except for a few species such as *Capsella bursa‐pastoris, T* *. pratense* , *Vaccinium myrtillus*, and *Potentilla erecta* (*p* > 0.05) in the ITS2 dataset.

**TABLE 3:** Results from a SIMPER analysis on the ITS2 dataset for the faecal DNA (*f*DNA) samples from the HD and LD diet treatments.

Taxon	Av. dissim	Contrib. %	Cumulative %	Mean HD *fDNA*	Mean LD *f*DNA	(ANOVA) *p*
*Lolium perenne*	15.68	19.89	52.7	40.4	10.6	6.33E−08*
*Trifolium repens*	13.15	16.68	69.38	25.6	0.00931	4.23266E−08*
*Alopecurus geniculatus*	1.695	2.151	80.45	3.35	0.0763	0.0001855*
*Cerastium glomeratum*	1.426	1.81	84.08	2.77	0	0.000603809*
*Holcus lanatus*	1.329	1.687	85.77	2.63	0.0399	0.001223141*
*Capsella bursa‐pastoris*	1.284	1.63	87.4	2.37	0.223	0.425761862
*Cerastium fontanum*	1.055	1.339	91.88	2.06	0.00832	1.20309E−06*
*Trifolium pratense*	0.9485	1.203	93.09	2.04	0.772	0.106043918
*Eriophorum vaginatum*	25.86	32.81	32.81	7.44	57.4	1.04153E−10*
*Avenella flexuosa*	4.711	5.977	75.36	0.914	9.85	7.17E‐05*
*Molinia caerulea*	2.313	2.935	78.29	0.439	4.56	0.005448861*
*Juncus squarrosus*	1.441	1.828	82.27	0.32	2.89	0.001599237*
*Vaccinium myrtillus*	1.26	1.599	89	0.46	2.39	0.018682827
*Potentilla erecta*	1.22	1.548	90.55	0.166	2.33	0.022280219

*Note:* Higher mean percentage values indicate that a species has a larger influence on the dissimilarity between diet treatments indicated by (green) most influential (yellow) second most influential. (*) indicates there is a significant difference.

**TABLE 4:** Results from a SIMPER analysis on the ITS2 dataset for LD vegetation and LD faecal (fDNA) samples.

Taxon	Av. dissim	Contrib. %	Cumulative %	Mean LD_*f*DNA	Mean LD_veg
*Eriophorum_vaginatum*	22.91	30.28	72.65	57.4	12.4
*Lolium_perenne*	5.334	7.049	79.7	10.6	0.13
*Avenella_flexuosa*	2.625	3.469	83.17	9.85	6.54
*Molinia_caerulea*	32.06	42.37	42.37	4.56	67.4
*Juncus_squarrosus*	1.468	1.94	88.01	2.89	0.0361
*Vaccinium_myrtillus*	1.102	1.456	94.81	2.39	0.398
*Potentilla_erecta*	1.202	1.589	93.35	2.33	0
*Urtica_dioica*	0.5941	0.7851	96.73	1.16	0
*Nardus_stricta*	2.197	2.903	86.07	0.342	4.34
*Galium_saxatile*	1.404	1.855	91.76	0.264	2.88

*Note:* Higher mean percentage values indicate that a species has a larger influence on the dissimilarity between diet treatments indicated by (green) most influential (yellow) second most influential.

For the LD diet, both markers displayed notable differences in species influence between vegetation and faecal samples. In the ITS2 dataset (Table 4), *M. caerulea* was the main contributor to dissimilarity in LD vegetation samples, whereas *E. vaginatum* and *L. perenne* dominated in the faecal samples. This contrasts with the botanical separations of the LD diet where the second most abundant species identified was *Festuca ovina* . In comparison to the trnL dataset (Table 5), the main taxa having an influence on the dissimilarity between diet treatments between the two contrasting diets are *T. repens* and *E. vaginatum* , followed by Poaceae species and *M. caerulea* . Similar to the ITS2 dataset, *E. vaginatum* stands out as having a very high average dissimilarity and a substantial contribution to the cumulative percentage, indicating its distinctiveness within the dataset.

**TABLE 5:** Results from a SIMPER analysis on the trnL dataset for the faecal DNA (*f*DNA) samples from the HD and LD diet treatments.

Taxon	Av. dissim	Contrib. %	Cumulative %	Mean HD_*f*DNA	Mean LD_*f*DNA	ANOVA *p*
*Trifolium repens*	21.58	21.91	66.67	43	0.0193	3.42E−06*
*Poaceae*	18.26	18.54	85.21	37.8	1.38	9.81058E−07*
*Trifolium pratense*	3.553	3.608	92.53	7.17	0.083	9.60812E−12*
*Holcus lanatus*	1.743	1.77	96.07	3.49	0.00983	0.001139584*
*Cerastium fontanum*	1.747	1.774	94.3	3.49	0.00656	0.032522162*
*Eriophorum vaginatum*	44.09	44.76	44.76	0	87.9	1.02927E−31*
*Molinia caerulea*	3.654	3.71	88.92	0	7.29	5.61817E−07*
*Agrostis vinealis*	0.6138	0.6232	96.7	1.22	0	0.001234499*
*Juncus bufonius*	0.6064	0.6157	97.31	0	1.21	0.000194682*
*Avenella flexuosa*	0.308	0.3128	98.85	0	0.614	0.000513718*

*Note:* Higher mean percentage values indicate that a species has a larger influence on the dissimilarity between diet treatments indicated by (green) most influential (yellow) second most influential. *p* results from an additional ANOVA test run on individual species to see if there is a significant difference between HD and LD diets, (*) indicates there is a significant difference.

**FIGURE 5:**
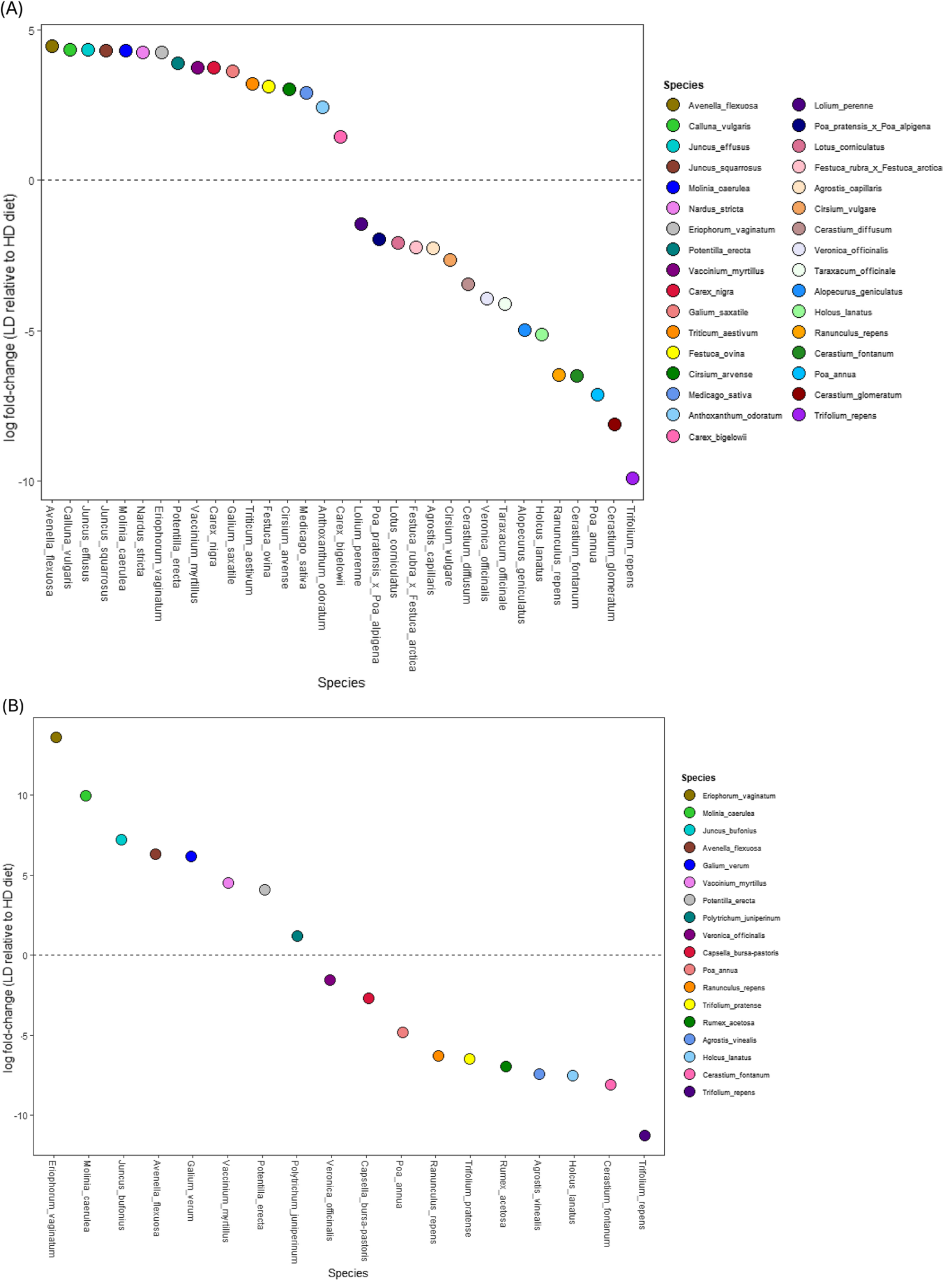
Log2fold differential abundance analysis of the different species between the HD and LD diets for the ITS2 dataset (A) and the trnL dataset (B). Differences were considered significant with *p*‐value (corrected for false positives using Benjamini‐Hochberg correction) at 0.05. The dashed 0 line is reflective of the baseline for HD diet. Points above this line are species that are significantly (*p* > 0.05) more abundant in the LD than the HD. So conversely, the species below this line are significantly less abundant in the LD diet compared to the HD samples. All matching ASVs were aggregated for this analysis so each dot represents a single species.

Differential abundance analysis further supported these trends, with 33 of 69 taxa (ITS2) (Figure 5A) and 18 of 45 taxa (trnL) (Figure 5B) showing significant differences (*p* < 0.05) between diets. In both markers, *E. vaginatum* maintained a high dissimilarity measure across samples, underscoring its prominence in the LD diet. The trnL dataset showed higher taxonomic aggregation, with Poaceae species often grouped at the family level, which limited species‐level distinctions compared to ITS2.

#### 3.1.5 Concluding Marker Comparison

Comparisons between the experimental diets (vegetation DNA and botanical composition) and corresponding faecal samples revealed marker‐specific patterns in dietary reconstruction. For both ITS2 and trnL, faecal samples from the HD diet closely reflected the composition of the experimental forage, with no significant divergence between faecal and vegetation samples (PERMANOVA, *p* > 0.05). In contrast, significant differences were observed between LD faecal and LD vegetation samples for both markers (*p*.adj < 0.015), indicating greater divergence between consumed and forage‐detected taxa under low‐digestibility conditions.

Species contributing most to these diet–faeces dissimilarities aligned with known differences in diet composition, with *Lolium perenne* and Trifolium spp. characterising the HD diet and *Eriophorum vaginatum* and *Molinia caerulea* contributing to the LD diet. While ITS2 provided higher species‐level resolution, particularly for legumes and forbs, trnL consistently detected dominant grass taxa but with reduced species specificity. Together, these results demonstrate that neither marker alone fully represents the experimental diet; instead, the combined use of ITS2 and trnL provides complementary information on taxonomic coverage and resolution when reconstructing herbivore diets from faecal DNA.

### 3.2 Trial 2—Known Diet Proportions

#### 3.2.1 Sequencing Data

Sequencing yielded 2,088,931 raw reads and 509 ASVs with a total of 48 taxa that comprised 17 families, 30 genera, and 33 species. The number of reads per ASV identified in the negative control samples was very low (< 50) compared to the samples (average 44,027), and these were removed from further analysis.

#### 3.2.2 Taxa Detection and Resolution

**FIGURE 6:**
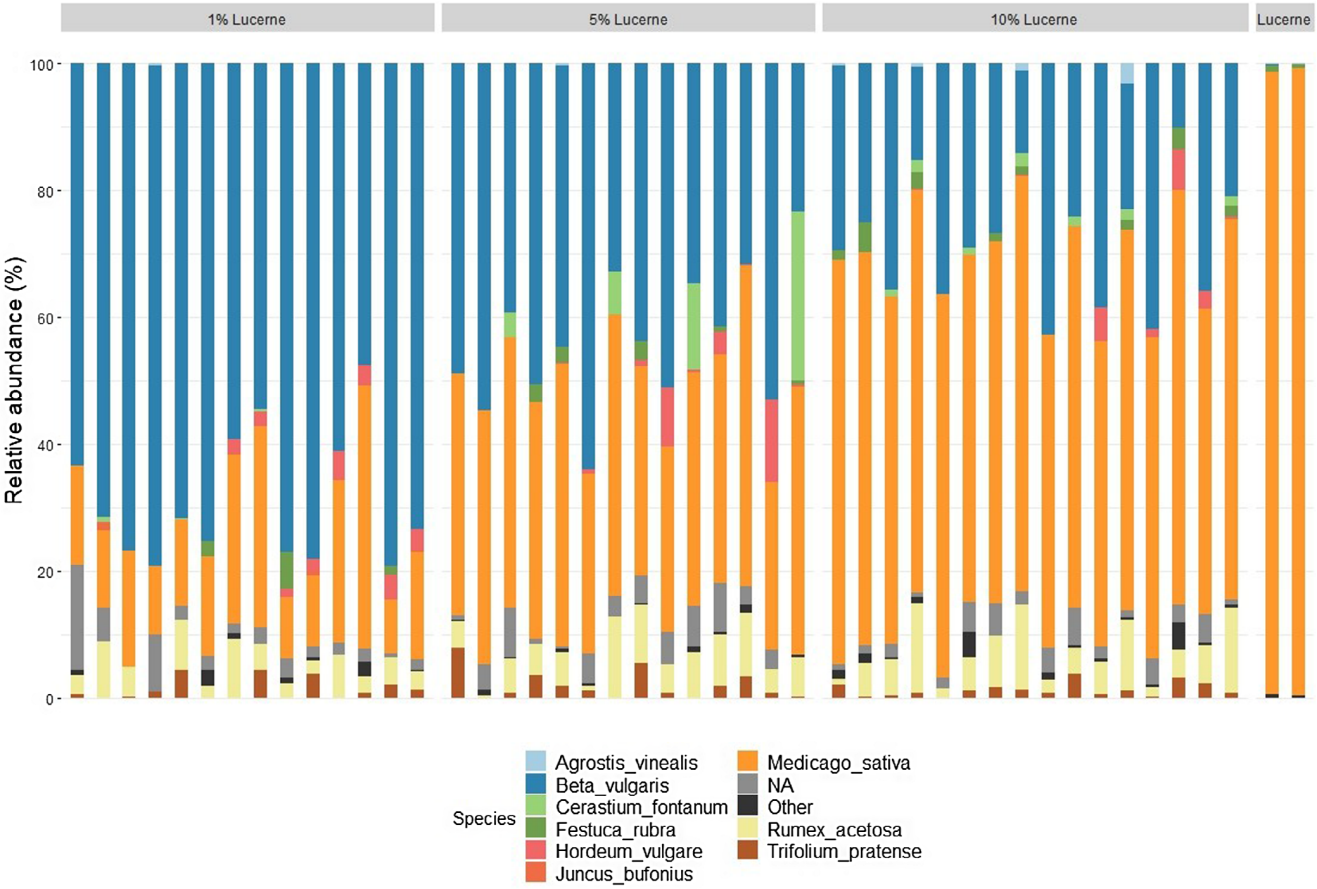
Taxa bar plot to show relative abundance (%) of species present within each diet treatment (1%, 5% and 10% of *M. sativa* addition) from the faecal DNA metabarcoding results, while ‘Lucerne’ represents the DNA samples from the pure *M. sativa* .

The taxa bar plot (Figure 6) indicates that *B. vulgaris* remains a major component of the diet throughout the treatments as expected. The spiked‐in species *M. sativa* was detectable as a dietary component as low as 1% and shows an increase in abundance from 1% to 5% and 10%, although the proportions of DNA sequences are over‐represented compared to the proportions added to the diet by weight (Figure 6). Relative abundances of *M. sativa* had a range of 2.8%–36.0% on the 1% diet, 29.3%–44.3% on the 5% diet, and 41.5%–58.0% on the 10% diet. Barley straw ( *H. vulgare* ) was a minor component of the basal diet but has not been detected in every faecal sample. Observations of animal behaviour during the trial recorded that individual sheep were observed selectively picking out and removing the straw feed to access the *B. vulgaris* and *M. sativa* material. Despite the simplicity of the diet in this feeding trial, the sequence data revealed a higher number of detected species than expected (Figure 6), which is likely due to some feed contamination as the botanical separations conducted on the bulked *M. sativa* harvested from the pasture also revealed the presence of *Lolium*, *Festuca*, *Rumex*, and *Trifolium* species.

#### 3.2.3 Dissimilarity and Major Taxa Contributors

The SIMPER analysis (Table 6) indicated that *M. sativa* and *B. vulgaris* were the primary contributors to dietary dissimilarity, accounting for 40.41% and 35.47% of the variance, respectively. *Medicago sativa* showed a progressive increase in presence from the 1% diet (16.10%) to the 10% diet (54.60%), while *B. vulgaris* exhibited a decreasing trend. Together, these two species accounted for 75.87% of the total observed dissimilarity among the diets, with other species contributing less significantly (16.15%) to the dissimilarity.

**TABLE 6:** Results from a SIMPER analysis for each diet treatment (1%, 5%, and 10%).

Species	Av. dissim	Contrib. %	Cumulative %	Mean 1% diet	Mean 5% diet	Mean 10% diet
*Medicago sativa*	13.02	40.41	40.41	16.10	37.40	54.60
*Beta vulgaris*	11.43	35.47	75.87	57.30	43.80	26.00
*Rumex acetosa*	2.24	6.94	82.81	3.54	5.86	6.14
*Hordeum vulgare*	1.48	4.60	93.95	1.33	2.05	1.01
*Trifolium pratense*	0.99	3.06	97.02	1.03	1.77	1.23
*Festuca rubra*	0.50	1.55	98.56	0.35	0.68	1.07

*Note:* The average dissimilarity (Av.dissim) value represents the average contribution of a particular species to the overall dissimilarity between diet treatment groups. Contribution percentage (Contrib %) indicates the proportion of the total dissimilarity between diet treatment groups that can be attributed to a specific species. The cumulative % value represents the running total of the percentage contributions of species to the overall dissimilarity (or similarity) between groups. Higher mean percentage values indicate that a species has a larger influence on the dissimilarity between diet treatments.

**FIGURE 7:**
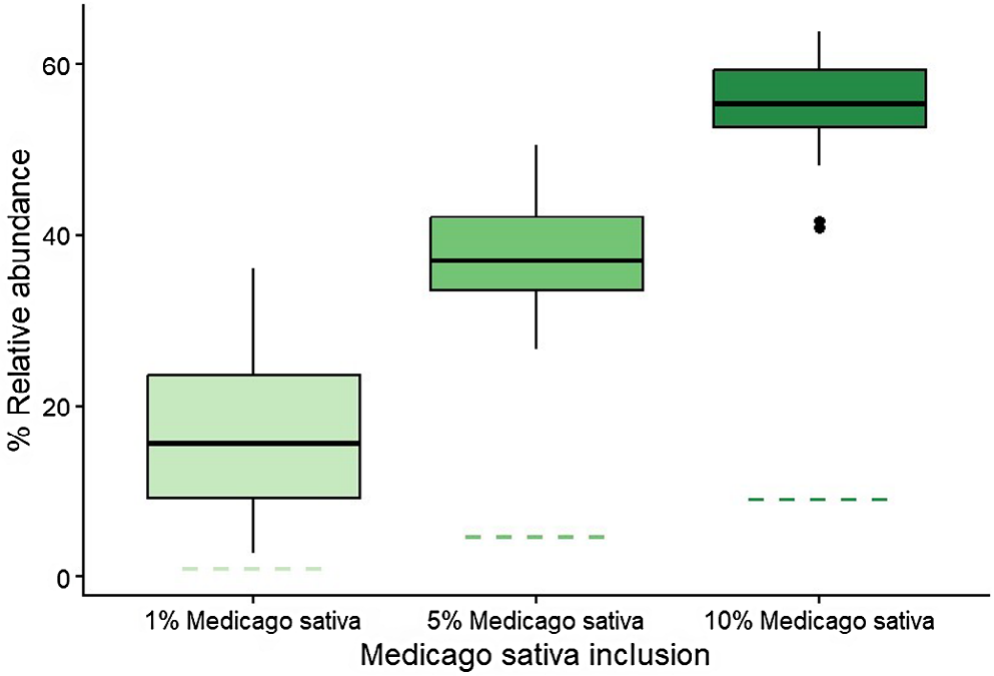
Box plot to show the relative abundance of *Medicago sativa* sequences recovered from faecal samples across three experimental diets (1%, 5% and 10% inclusion). Coloured boxplots show the distribution of sequence relative abundance among samples for each diet. Horizontal dashed lines indicate the expected dietary proportion of *Medicago sativa* in the as‐fed ration.

A Shapiro–Wilk test confirmed that the data were not normally distributed (*p* < 0.01). Differential abundance analysis further showed significant differences in the abundance of the main dietary components across the three diets (*p* < 0.001), with pairwise comparisons identifying significant differences in *M. sativa* and *B. vulgaris* between the 1% and 10% diets but not between 1% and 5% or between 5% and 10% (Table 7). *Medicago sativa* was notably overrepresented in sequence data relative to its dietary proportion, with a general correlation observed between expected and sequenced proportions, albeit *much higher than expected from fed amounts* (Figure 7).

**TABLE 7:** Table of results from a differential abundance analysis with a specific contrast between diet treatments 1% and 5%, 1% and 10%, 5% and 10%, respectively.

Treatment	Species	Basemean	Log2foldchange	lfcse	Stat	*p*	*p*_adj_
1% vs. 5%	*Medicago Medica*	23746.513	−0.889516	0.146431	0	1	1
	*Beta Vulgaris*	11587.435	0.96054	0.137216	0	1	1
	*Hordeum vulgare*	391.347	−0.212457	1.763919	0	1	1
1% vs. 10%	*Medicago Medica*	23746.513	−1.583112	0.141796	−4.11234	3.92E−05	5.88E−05*
	*Beta Vulgaris*	11587.435	1.62065	0.13293	4.66901	3.03E−06	9.08E−06*
	*Hordeum vulgare*	391.347	0.602349	1.708045	0	1.00E+00	1.00E+00
5% vs. 10%	*Medicago Medica*	23746.513	−0.693596	0.141665	0	1	1
	*Beta Vulgaris*	11587.435	0.660111	0.132926	0	1	1
	*Hordeum vulgare*	391.347	0.814806	1.707955	0	1	1

*Note:* * indicates value is significant.

## 4 Discussion

This study evaluated the accuracy of DNA metabarcoding in estimating the plant species composition of forage diets fed to sheep under controlled conditions, using two contrasting diets (high and low digestibility) and two genetic markers (ITS2 and trnL) to assess biases in detecting major and minor dietary components. Both markers effectively distinguished between the HD diet, primarily composed of *L. perenne* , Poaceae species, and *T. repens* , and the LD diet, which was dominated by *E. vaginatum* and *M. caerulea* . Notably, ITS2 identified 56 species compared to 31 identified by trnL, with 22 species shared between both markers. ITS2 provided higher species‐level resolution, whereas trnL captured broader family‐level classifications. This complementarity highlights the utility of using both markers to reconstruct complex herbivore diets while also revealing marker‐specific biases. In a further experiment, DNA metabarcoding was applied to diets with specific inclusion levels (1%, 5%, and 10%) of the target species *M. sativa* to explore detection and quantification reliability. *Medicago sativa* was detectable when present at as low as 1%, suggesting that DNA metabarcoding can reliably identify minor dietary species—a valuable capability for dietary studies where understanding trace components is essential (Stapleton et al. 2022). Sequence reads of *M. sativa* also increased with larger amounts included in the diet, although the target species was consistently overrepresented in the sequence data relative to its inclusion proportion.

In the digestibility trial, trnL sequences had fewer species‐level assignments compared to ITS2. At the genus level, trnL classified these sequences as *Festuca* species, whereas ITS2 indicated the presence of *L. perenne* . This is likely due to the close phylogenetic relationship between these taxa, which belong to the *Festuca–Lolium* complex, a group characterised by recent divergence and extensive hybridisation, resulting in high sequence similarity across commonly used DNA barcode regions (Cheng et al. 2015; Soreng et al. 2022; Watson 1990). This highlights a broader challenge in using DNA metabarcoding to resolve herbivore diets composed of closely related grass species. Furthermore, the prevalence of hybridisation within this complex means that metabarcoding markers may detect signatures of multiple parental species or may be unable to distinguish hybrids from their parental taxa, making it difficult or impossible to confidently identify hybrid species in herbivore diets.

While ITS2 provided higher overall taxonomic resolution, clearly identifying *Lolium perenne* and *Trifolium* spp. as dominant components of the high‐digestibility (HD) diet, it also showed some discrepancies with botanical separations in the low‐digestibility (LD) diet, indicating that misassignments or incomplete reference coverage remain possible. In contrast, trnL generated a higher proportion of ASVs that could not be resolved beyond broad ranks (e.g., Poaceae or Streptophyta), limiting species‐level identification within grasses. Our results therefore suggest that ITS2 performs better than trnL for resolving Poaceae in this system, but not necessarily for all plant families, and that marker performance is both taxon‐ and context‐dependent.

This reflects a wider challenge with “universal” metabarcoding markers. It is inherently difficult to design primers and barcode regions that offer both broad taxonomic coverage and fine‐scale resolution, and when using the trnL P6 loop some groups, including Poaceae, are known to be poorly resolved (Taberlet et al. 2018; De Barba et al. 2014). In this study, we used a standard naïve Bayesian classifier and a region‐restricted reference database to mirror common herbivore‐diet workflows, but our findings highlight that taxonomic assignments are shaped jointly by marker choice, reference completeness, and classifier settings (da Silva et al. 2019). Future work combining ITS2 with family‐specific primers, or adopting higher‐resolution sequencing strategies, may help address these limitations. Shotgun metagenomics offers the potential to recover multiple genomic regions or entire organelle genomes, reducing reliance on a single barcode region and improving discrimination within taxonomically challenging families such as Poaceae (Chua et al. 2021).

Across both trials, there was a discrepancy between sequence read relative abundance and actual dietary proportions, particularly noticeable in the faecal samples. Species' proportions from digestibility vegetation diet mixtures were more accurately reflected in the sequencing data for the vegetation samples than from the faecal samples, supporting previous observations that differential digestibility can impact DNA survival through the digestive tract and introduce biases in the metabarcoding analysis (Nichols et al. 2016; Scasta et al. 2019). The recovery of plant DNA from faecal samples is influenced not only by the amount of material that passes through the digestive system, but also by how much of that material is broken down during digestion. Highly digestible plant species are more thoroughly degraded in the gut, which can result in lower amounts of intact plant DNA being recovered in faeces. In contrast, more fibrous or poorly digestible plant tissues (e.g., stems, lignified tissues) pass through the digestive tract with greater structural integrity, leading to higher recoverable DNA levels (Neidel and Traugott 2023; Stapleton et al. 2022). These patterns are further shaped by factors such as the herbivore's digestive physiology, gut retention time, and microbiome activity, all of which influence how much intact plant DNA persists through digestion (Kartzinel et al. 2015). In the LD trial, both markers showed dominance of *M. caerulea* in the vegetation samples but *E. vaginatum* in the faecal samples, which may be due to species‐specific biases in diet selection or digestion. This could be due to the nature of the plant tissue samples and the ease of extracting amplifiable DNA. Low digestibility forage (such as *Juncus*, *Molinia* and *Eriophorum*) typically has higher lignin content providing structural support to these more woody or fibrous plants (Armstrong et al. 1986; Grossi et al. 2019). Forage with a higher lignin content not only reduces the digestibility and nutritional value of mature plant material but is also correlated with a higher content of phenolic compounds (Varma et al. 2007; Lee 2018). Such secondary metabolites are reported to inhibit DNA extraction process and further affect the yield and purity of DNA for downstream processing which could be influencing the sequence results (Rucińska et al. 2021).

In the proportions trial *M. sativa* was consistently overrepresented in the sequence data relative to its inclusion rate, aligning with prior findings where target species tend to be overestimated in sequence counts due to factors such as differences in chloroplast DNA quantity per gram of tissue, differential digestibility, and PCR amplification biases (e.g., primer affinity) (Stapleton et al. 2022; Willerslev et al. 2014). These findings align with past research evidence from captive animal feeding trials, which have also struggled to achieve quantitative accuracy when comparing sequenced data to known dietary inputs (Deagle and Tollit 2007; Deagle et al. 2010; Bowles et al. 2011; Thomas et al. 2014). For instance, in a similar controlled feeding study with woodrats by Stapleton et al. (2022), overestimation of *Juniperus osteosperma* and *Larrea tridentata* was observed, suggesting that differential amplification and degradation rates among plant species can skew quantification results (Stapleton et al. 2022). A study on *Cervus nippon* (sika deer) showed DNA metabarcoding could detect all species fed, but discrepancies between sequenced and consumed plant proportions were linked to the complex digestive processes of herbivores (Nakahara et al. 2016). Other faecal DNA metabarcoding studies on a wide range of species ( *Phoca vitulina* (harbour seals), *Eudyptula minor* (little penguins), *Gallus gallus* (chickens), *Acinonyx jubatus* (cheetahs)) similarly found variability between relative read abundance and consumed food, suggesting that quantitative DNA data should be interpreted cautiously (Deagle et al. 2010, 2013; Thuo et al. 2019; Thongjued et al. 2024). Collectively, these studies highlight that while DNA metabarcoding is highly effective for detecting dietary species presence, inferring absolute or proportional biomass consumption from sequence read counts remains challenging. This is particularly evident for taxa that are either over‐ or under‐represented during digestion, amplification, or sequencing, potentially biasing the detection of both major and minor dietary components.

Nevertheless, despite this longstanding debate, several studies have identified encouraging trends toward more quantitative interpretations of DNA metabarcoding data when biological and technical biases are explicitly addressed. Krehenwinkel et al. (2017), Piñol et al. (2018), and Stapleton et al. (2022) suggest that improved experimental design, marker selection, reference database quality, and bioinformatic processing can enhance the relationship between sequence reads and dietary proportions. These findings indicate that, although DNA metabarcoding may not provide precise estimates of biomass consumed, it can reliably capture relative differences in dietary composition among samples and treatments when applied within a controlled and well‐characterised framework.

One explanation for discordant proportions is primer bias. To investigate this for our study, we mapped the *trn*L primers to the chloroplast genomes of *B. vulgaris* , *H. vulgare* , and *M. sativa* . *Hordeum vulgare* showed no mismatches, while *M. sativa* and *B. vulgaris* each had a single mismatch (to trnL‐c and trnL‐h, respectively), both located toward the 5′ end of the primer. Such mismatches are unlikely to substantially affect primer binding efficiency and therefore do not explain the consistent overrepresentation of *M. sativa* relative to the other dietary components. This suggests that additional factors beyond primer template mismatches such as differences in template abundance, DNA quality or amplification efficiency are likely contributing to the observed bias. Additionally, chloroplast content differences among dietary components could play a crucial role. As a green leafy plant, *M. sativa* has a high chloroplast count, essential for photosynthesis, whereas *H. vulgare* and *B. vulgaris* —mainly composed of stems, leaves, and roots—contain relatively fewer chloroplasts. This discrepancy may lead to a higher abundance of *M. sativa* DNA during PCR when using a chloroplast marker, thus inflating its apparent abundance in sequencing data (Stapleton et al. 2022). Research underscores that primer choice and cell copy number significantly impact amplification bias, with certain markers favouring specific taxa (Taberlet et al. 2007; Garnick et al. 2018; Deagle et al. 2019). With regards to the proportions trial, using only the chloroplast marker trnL may have amplified *M. sativa* disproportionately, given its high chloroplast DNA content, resulting in skewed proportions (Valentini, Pompanon, and Taberlet 2009; Soininen et al. 2015). This reinforces the importance of employing multiple complementary markers to reduce bias and achieve a more accurate dietary composition in complex herbivore diets (Pompanon et al. 2012; Goldberg et al. 2020).

Mallott et al. (2018) states that “controlled feeding studies where the amount of a food consumed and gut passage rates are known are imperative to more precisely determine whether or not relative abundances can accurately be used to quantify the relative contributions of plants foods to animal diets” (Mallott et al. 2018). Despite the advancement of DNA metabarcoding for dietary studies, little research has been carried out (particularly for herbivores) to fully understand how digestion and DNA survival can influence reconstructing diets. In feeding trials on sea lions, DNA from different prey species was found to degrade at different rates, leading to biased recovery in faecal samples that did not reflect the actual proportions consumed (Deagle et al. 2013; Deagle and Tollit 2007). Similarly, Nakahara et al. (2016) used captive deer to compare known dietary inputs with faecal metabarcoding results and found that certain plant species were consistently underrepresented in sequence data, suggesting differential digestion or amplification biases. These findings suggest that there are distinct biases in the preservation of DNA from different prey/plant species during the process of digestion, and that faecal proportions did not match those in the consumed tissue mixes. Deagle goes on to suggest that more accurate species‐specific correction factors and calibration studies (e.g., Yoccoz et al. 2012) are required to take into account differential digestion if accurate quantification of diets is to be achieved (Shelton et al. 2023).

Although the proportion diet contained only three components, DNA analysis revealed a higher‐than‐expected number of species, likely due to contamination from surrounding vegetation and straw. This contamination potentially arose due to the direct harvesting of *M. sativa* from experimental plots sown with a single species but likely including contamination from the seed bank and surrounding areas. It is also plausible that additional species were imported with the straw material.

In this study, diet composition was assessed using faecal DNA only; however, other studies have also examined stomach contents. Comparisons between these two sample types in passerines found that both yielded comparable results in terms of dietary richness and diversity, although DNA concentrations were typically higher and more consistent in stomach contents (Snider et al. 2022). Nonetheless, each sample type provides information over different temporal scales; stomach contents reflect recent feeding events, whereas faecal samples can integrate diet over a longer period and may include more digested material. Consequently, both approaches have inherent biases and limitations: stomach contents can overrepresent recently consumed prey, while faecal DNA can vary in detectability due to digestion and retention time. These differences highlight the importance of interpreting dietary proportions with caution and developing integrative approaches that account for both biological and methodological sources of variation. Using different genetic markers (mitochondrial, nuclear, chloroplast) in DNA metabarcoding greatly influences study outcomes, particularly for dietary analysis. Plant studies typically avoid mitochondrial markers due to their slow mutation rate, which means there is less genetic variation to distinguish between different plant species (Hollingsworth et al. 2011; Guo et al. 2022; Zhu et al. 2022). Instead, the use of chloroplast markers such as rbcL, matK, and *trn*L (as in this study) is preferred for plant barcoding. These chloroplast genes provide higher resolution for species identification and are more amenable to standardisation across a wide range of plant taxa (Zhu et al. 2022). However, to mitigate the bias introduced by potential differences in chloroplast content (especially for herbivorous diets), researchers advocate that an effective way to improve the accuracy and taxonomic resolution for plant barcoding is to use a combination of multiple barcoding regions that complement each other instead of relying on a single marker (Ando et al. 2020). For example, nuclear DNA regions like ITS may provide a more reliable and unbiased representation of species proportions in the sampled material (Chen et al. 2010). These two markers (*trn*L and ITS2) are now becoming the most used for plant barcoding (Guo et al. 2022). Adopting a multi‐marker approach in future studies could provide complementary information and act as a cross‐verification of results to achieve a more comprehensive picture of the species composition within a diet. In addition, developing marker‐specific correction factors could help account for systematic biases such as differences in chloroplast and nuclear DNA copy numbers or amplification efficiencies. While such corrections would not fully eliminate quantitative distortions caused by digestion or sequencing depth, they could improve comparability between markers and enhance the accuracy of relative abundance estimates in dietary metabarcoding studies. A major challenge in metabarcoding is achieving quantitative accuracy due to differences in measurement methods; dietary input is dry weight‐based, while metabarcoding relies on DNA quantity. Quantitative accuracy might be enhanced by incorporating mock vegetation mixtures at specific proportions as calibration references to identify any sequencing noise. Despite these challenges, trends observed in faecal DNA, such as the increase in *M. sativa* sequences with its increased dietary presence, highlight the method's utility in tracking dietary shifts, even if exact input proportions are not directly mirrored (Deagle et al. 2019; Zinger et al. 2019).

## 5 Conclusion

This study integrates results from two controlled sheep diet trials to evaluate the effectiveness of DNA metabarcoding in estimating plant species composition under contrasting high‐ and low‐digestibility diets, and in detecting a target species, using the ITS2 and *trn*L markers. Findings from both trials indicate that DNA metabarcoding is a promising tool for characterising plant‐herbivore interactions, capturing dietary composition, and differentiating diet‐associated plant communities. However, limitations emerged, particularly in accurately identifying species within the Poaceae family, suggesting that closely related species may require alternative markers. Challenges were also noted in distinguishing faecal and vegetation samples for the low digestibility diet, potentially due to factors such as plant tissue type, differential digestibility, and DNA degradation through the digestive tract.

A key insight from these trials is the potential for DNA metabarcoding to detect target species like *M. sativa* at varying and very low inclusion levels. However, *M. sativa* was consistently overrepresented in the DNA results, likely due to primer specificity and the high chloroplast content in this species. This overrepresentation underscores the need to consider primer choice carefully, particularly for chloroplast markers like trnL, which may appear more abundant in species with higher chloroplast content. Employing a multi‐marker approach, incorporating both chloroplast and nuclear DNA regions, could provide a more balanced representation of dietary components. Additionally, introducing mock vegetation mixtures and control diets in experimental designs would improve calibration and enhance accuracy in dietary quantification.

Overall, while DNA metabarcoding demonstrates clear potential for dietary analysis, its quantitative accuracy remains challenging, influenced by primer bias and dietary component characteristics. Future research should focus on multi‐marker strategies, optimised bioinformatic pipelines, and methodological improvements to address these limitations, thereby enhancing the resolution and reliability of metabarcoding in herbivore diet studies. Despite these challenges, the ability of faecal DNA metabarcoding to capture dietary trends and indicate dietary preferences offers valuable insights into herbivore diet composition and environmental interactions.

## Author Contributions

**Hannah Vallin:** conceptualization (equal), data curation (lead), formal analysis (equal), methodology (equal), writing – original draft (lead), writing – review and editing (equal). **Mariecia Fraser:** conceptualization (equal), funding acquisition (supporting), methodology (equal), supervision (equal), writing – review and editing (equal). **Robin J. Pakeman:** funding acquisition (supporting), writing – review and editing (equal). **Helen Hipperson:** conceptualization (equal), formal analysis (equal), supervision (equal), writing – review and editing (equal).

## Funding

This work was supported by the Natural Environment Research Council, NE/R016801/1; Biotechnology and Biological Sciences Research Council, BBS/E/IB/230001A.

## Conflicts of Interest

The authors declare no conflicts of interest.

## Supporting information


**Data S1:** ece372878‐sup‐0001‐DataS1.docx.

## Data Availability

Outputs will be freely available on Aberystwyth University's publicly available online repository, PURE https://doi.org/10.20391/588897d4‐9e4c‐4c96‐97cf‐4047b4ca1eb2.
