# Supplementary material for: Evaluating the Quantitative Accuracy and Application of DNA Metabarcoding for Dietary Reconstruction in Ruminants
Source: Ecol Evol. 2026 Jan 14;16(1):e72878. doi: 10.1002/ece3.72878 (PMC12801136; doi:10.1002/ece3.72878)
Supplement: Supplementary file 1 — Data S1: ece372878‐sup‐0001‐DataS1.docx. [file ECE3-16-e72878-s001.docx]

Supplementary Information

## Trial one experimental procedure:

The grass forage and legume mixture (Diet 1: high digestibility) was cut directly from controlled experimental pastures at Pwllpeiran (230 meters above sea level) using a Gianni Ferrari mower and stored in open mesh-sided baskets within a cool room at approximately 4 °C. Previous research suggests that forage material can be stored in this way for up to 5 d without spoilage (Fraser et al., 2004). Assuming an estimated intake of 1 kg dry matter (DM) per head per day for the 5d experimental period, and a forage DM content of 20%, a minimum quantity of 100 kg of fresh forage was required. The fresh forage from this site was predominantly a *Lolium spp.* (ryegrass) and *Trifolium spp.* (clover) mix that had been sown in 2018, with some contamination from the natural seed bank. The hill grass *Molinia* mix (Diet 2: Low digestibility) was cut from accessible semi-natural grassland on the Fridd/Garn area at Pwllpeiran (540 meters above sea level) using a finger-bar mower. Following collection, this was also stored in baskets and chilled as above. Assuming an estimated intake of 1 kg DM per head per day for the 5d experimental period, and a forage DM content of 40%, a minimum quantity of 50 kg of fresh forage was required for the trial.

Dry matter (DM) content will be estimated on the day of cutting by weighing triplicate 200 g sub-samples of forage, oven drying the samples overnight at 80 °C, and re-weighing. Each feed offered will be weighed out in accordance to the individuals’ maintenance requirements and the DM content of the forage, and recorded on a daily basis. Any refusals will be collected, weighted and retained for further analysis.

As the experimental diets are being measured out a sub-sample of around 200 g will be collected daily and bulked across the 5 d experimental period. Following thorough mixing, a 100 g sample of this bulked material will be undergo botanical separation into component plant species carried out by a trained botanist following identification keys from Hubbard (1954) and Fitter, R., Fitter, A. & Blamey, M. (1974). The separated material will be oven dried to constant weight at 40 °C, weighed, and the proportional botanical composition of the forage as fed expressed on a DM basis. Any refusals will be separated in a similar way.

For the initial adaptation phase (8d) of the experiment the animals were brought into a group pen in the sheep shed and allowed to acclimatise to being in a housed environment. During this time, hay was fed *ad libitum* and grass pellets (500 g) were provided twice a day am and pm (i.e. a mix of both high and low digestibility feeds were offered). The amount of grass pellets fed during the adaptation phase was calculated based upon the average maintenance requirement value. Following 4d within a group pen the sheep were transferred to adjacent individual pens for a further 4d, allowing time to adjust to being separated from conspecifics before the experimental diet began. Fresh water and bedding were provided daily and cleaned as needed throughout the entire experiment. Once individually penned and for the remainder of the experimental period (5d) all animals were fed to maintenance requirements according to Agricultural Food and Research Council (AFRC 1993). The forage quantities for diets 1 & 2 required were calculated for each individual based on their body weight and fed in two equal portions twice a day at 0900 and 1600. All experimental animals were wethers 2+years of age, therefore they do not require additional feeding and can be fed to body maintenance as their physiological state is stable, they are not growing lambs nor lactating ewes with higher energy demands. Feeding to maintenance also ensures that all the forage will be consumed and further minimises any risk of selective feeding biasing the results. Following completion of the first experimental diet (1) trial the sheep were then returned to the adaptation phase and fed only hay *ad libitum* in preparation for the low digestibility diet run with the hill grass mixture (Diet 2). During each experimental run the animals were housed in individual pens and bedded on rubber mats (to enable faecal collection and ensure bedding material was not consumed).

Fitter, R., Fitter, A. & Blamey, M. (1974). The Wild Flowers of Britain and Northern Europe. London: Collins.

Hubbard, C.E. (1954). Grasses. Rev. ed. by J.C.E. Hubbard, 1984. London: Penguin.

## Trial two experimental procedure:

The same cohort of twelve adult Soay wethers (>2 years old) used in Trial 1 was subsequently used for Trial 2 under identical housing and welfare conditions at the Pwllpeiran Research Centre. Prior to start of the experiment the sheep were pastured together on long-term improved permanent pasture which contains a low proportion of ryegrass (Lolium spp.) and white clover (Trifolium repens), as well as a variety of unsown grass, forb, and rush species. At least 5 days prior to bringing the sheep in, small portions of B. vulgaris were offered in field to help ensure a gradual rumen microbial adaptation to the diets subsequently offered.

During the initial adaptation period all Soay sheep were housed in a group pen, with fresh water and bedding supplied at all times during the whole experiment. The adaptation period lasted for 14 days during which they were fed the basal diet ad lib. A long adaption period was required for the start of this experiment as there was a significant change in diet from the grazed pasture site to the experimental diet.

After the adaptation period, each animal was placed in an individual pen with sawdust bedding. Over the course of 6 days, all animals were exclusively provided with the basal diet. During this phase, the feed was adjusted to meet each animal's specific maintenance requirements as outlined by AFRC (1993) guidelines. The quantities required were calculated for each individual based on their body weight and fed in two equal portions twice daily (08:00, and 16:00). This extended period of basal diet feeding was to allow adaptation to being individually penned in order to minimise any distress and maintain the welfare of all individuals.

The experimental diet period lasted 5 days, with faecal sampling carried out during the last two days. During the experimental diet period individuals were allocated to one of the three experimental diets (1%, 5% or 10%), all running simultaneously for 5 d. Sheep were ranked in ascending order by weight and allocated to one of the experimental diets, to ensure there was no individual weight bias influencing results. As with the basal diet, all experimental diets were fed to maintenance requirements for each individual. At the end of the trial period all sheep were re-weighed, and condition scored before moving back to pasture.

## Two step PCR

The complete second internal transcribed spacer of nuclear ribosomal DNA (ITS2) and the P6 loop of the plastid trnL (UAA) region were amplified across all samples following a two-step PCR. Mock community samples were also added into the PCRs and sequencing to act as a quality control measure to help assess the accuracy and reliability of sequencing results. These samples comprised of 5 known tropical plant species from pre-existing DNA extracts, (supplied from The National Botanic Garden of Wales) (Supplementary information 5).

Table 1. Sequences of the two universal primer pairs used to amplify the ITS2 and trnL regions.

| **Gene** | **Primer name** | **Primer (5'-3')** | **Primmer source** |
| --- | --- | --- | --- |
| ITS2 | UniPlantR | CCCGHYTGAYYTGRGGTCDC | Moorhouse-Gann et al., (2018) |
| ITS2 | ITS2F | ATGCGATACTTGGTGTGAAT | Chen et al., (2010) |
| trnL | trnLc | CGAAATCGGTAGACGCTACG | Taberlet et al., (2007) |
| trnL | trnLh | CCATTGAGTCTCTGCACCTATC | Taberlet et al., (2007) |

The first PCR amplified the gene region using amplicon-specific primers (Table 1) tailed with generic adapters. The PCR was carried out in a 20 μl reaction volume using 2 μl of template DNA (at a 1:2 dilution), 10 μl of PCR master mix (Qiagen Multiplex PCR Master Mix (2x)), 2 μl each of the forward and reverse primers (at 1 to 5 μM), and 4 μl of sterile double-distilled water (ddH2O). Negative controls were added for every PCR to verify no contamination had been introduced. Thermocycling conditions for the first PCR were: 95 °c for 15 min, followed by 40 cycles of 95 °C for 30 s, 56 °C for 30 s, 72 °C for 60 s, and then 72 °C for 10 min. PCR products (4 μl) were run on a 1% agarose gel to ensure successful amplification and that minimal primer dimer was present. Following this, purification of the PCR product was carried out with ProNex beads (Promega) following the manufacturer’s protocol using a 1.5X bead:DNA ratio and eluted in 15 μl of low Tris-EDTA buffer.

The second PCR was carried out to add on unique index sequences to each sample (dual-indexed: Fi5 and Ri7 primers in unique combination for each sample) and the Illumina p5 and p7 sequences to the amplicon products. PCRs were carried out in 20 μl reaction volumes using 8 μl of template product from PCR1, 10 μl PCR master mix (Qiagen Multiplex PCR Master Mix (2x)), 1 μl of Fi5/Ri7 primers (at 10 μM), and 1ul ddH2O. PCR conditions were: 95 °C for 15 min, followed by 10 cycles of 98 °C for 10 sec, 65 °C for 30 sec, 72 °C for 30 sec, then 72 °C for 5 min.

PCR2 products were quantified using fluorometry and 150ng from each of 8 samples pooled for bead cleaning. The pooled samples were purified using ProNex beads (Promega) following the manufacturer’s protocol with a 1.5X bead:DNA ratio and a final elution volume of 15μl low Tris-EDTA buffer. qPCR was carried out on each cleaned pool using KAPA library quantification kit following the manufacturer’s protocol. Concentrations from qPCR were then used for equimolar pooling of all samples and to dilute the library to the desired concentration (5nM) for sending for sequencing. Pre- and post-PCR2 products were also run on the Tapestation system (Agilent 4200) to ensure the absence of primer dimer and check products were of the expected length. The libraries were sequenced at Edinburgh Genomics (University of Edinburgh, Edinburgh, UK) using Illumina MiSeq v2 500 cycle kits.

## Barcode and adaptor sequences

****Barcode and adaptor sequence information for the 12 forward and 8 reverse trnL barcodes.

## Mock community species

List of tropical mock community species provided from The National Botanic Garden of Wales. These comprised equal proportions of DNA from five known tropical plant species sourced from pre-existing DNA extracts supplied by the National Botanic Garden of Wales.

| **Phylum** | **Class** | **Order** | **Family** | **Genus** | **Species** |
| --- | --- | --- | --- | --- | --- |
| Streptophyta | Magnoliopsida | Dilleniales | Dilleniaceae | Dillenia | Dillenia excelsa |
| Spermatophyta | Magnoliopsida | Ericales | Sapotaceae | Madhuca | Madhuca-dubardii |
| Magnoliophyta | Magnoliopsida | Gentianales | Rubiaceae | Urophyllum | Urophyllum-griffithianum |
| Streptophyta | Magnoliopsida | Malvales | Malvaceae | Kleinhovia | Kleinhovia-hospita |
| Phyllanthaceae | Magnoliopsida | Malpighiales | Phyllanthaceae | Antidesma | *Antidesma thwaitesianum* |
